# Tempol improves optic nerve histopathology and ultrastructures in cisplatin-induced optic neuropathy in rats by targeting oxidative stress—Endoplasmic reticulum stress—Autophagy signaling pathways

**DOI:** 10.3389/fncel.2023.1256299

**Published:** 2023-10-05

**Authors:** Amira Ebrahim Alsemeh, Mohey A. E. Hulail, Hanan E. L. Mokhtar, Reham Talaat Eldemerdash, Ioan Banatean-Dunea, Liana Mihaela Fericean, Maha Abdelhamid Fathy, Ahmed Hamed Arisha, Tarek Khamis

**Affiliations:** ^1^Human Anatomy and Embryology Department, Faculty of Medicine, Zagazig University Egypt, Zagazig, Egypt; ^2^Department of Biology, Faculty of Agriculture, University of Life Sciences, King Mihai I” from Timisoara [ULST], Timisoara, Romania; ^3^Medical Physiology Department, Faculty of Medicine, Zagazig University, Zagazig, Egypt; ^4^Department of Animal Physiology and Biochemistry, Faculty of Veterinary Medicine, Badr University in Cairo, Badr City, Egypt; ^5^Department of Physiology, Faculty of Veterinary Medicine, Zagazig University, Zagazig, Egypt; ^6^Department of Pharmacology, Faculty of Veterinary Medicine, Zagazig University, Zagazig, Egypt; ^7^Laboratory of Biotechnology, Faculty of Veterinary Medicine, Zagazig University, Zagazig, Egypt

**Keywords:** optic neuropathy, autophagy, endoplasmic reticulum stress, tempol, cisplatin (Cis)

## Abstract

**Introduction:**

Optic neuropathy is an affection of the optic neurons, which ends with blindness and occurs either primarily due to direct affection of the optic nerve or secondarily as a complication of chronic diseases and/or adverse effects of their therapy. The search for novel therapeutic tools is crucial in addressing the limited therapeutic approaches for optic neuropathy. Therefore, the present study was developed to investigate the possible ameliorative effect of tempol against cisplatin-induced optic neuropathy and its underlying mechanism.

**Methods:**

Forty-eight adult male albino Wistar rats were divided into four equal groups—control, tempol (TEM), cisplatin (CIS), and tempol and cisplatin combined (TEM+CIS). Optic nerve oxidative stress (MDA, SOD, and GPx), gene expression of endoplasmic reticulum stress (*ATF-6, XBP-1, BIP, CHOP*, and *JNK*), autophagy 6 (LC3, Beclin-1, and p62) markers, nerve growth factor-1, immunohistochemical expression of (LC3 and p62), histopathological, and electron microscopic examination were performed.

**Results:**

Histopathological and ultrastructure examination validated that cisplatin caused optic neuropathy by inducing oxidative stress, upregulating ER stress markers, and downregulating autophagy markers, and NGF-1 expression. TEM + CIS showed improvement in optic nerve structure and ultrastructure along with oxidative stress, ER stress mRNA, autophagy (immunohistochemical proteins and mRNA) markers, and nerve growth factor mRNA expression.

**Conclusions:**

Based on previous findings, tempol represents a valid aid in cisplatin-induced optic neuropathy by implicating new molecular drug targets (ER stress and autophagy) for optic neuropathy therapy.

## 1. Introduction

Tempol is a synthetic, low molecular weight, water-soluble, cyclic nitroxide, and membrane-permeable superoxide dismutase mimetic. It is reported to be an effective antioxidant and radical scavenger (Da Silva et al., 2020). Tempol can preserve mitochondria against oxidative damage and enhance tissue oxygenation. It also reduces lipid peroxidation, tyrosine nitration, protein carbonyl formation, and DNA damage (Wilcox, 2010). Tempol is capable of penetrating the blood-brain barrier easily and yielding neuroprotective effects because of its low molecular weight (Youn et al., 2016). Tempol can inhibit the activation of NF-κB and downstream pro-inflammatory gene expression, thereby exerting anti-inflammatory effects (Francischetti et al., 2014). Cisplatin is an extremely effective chemotherapeutic agent and is widely used to treat a variety of malignant tumors in the ovary, endometrium, head, neck, breast, pulmonary, bladder, testicular, and colorectal cancer (Quintanilha et al., 2019). The antineoplastic action of cisplatin is exerted by binding directly to DNA and forming crosslinks that interfere with cellular functions, thus leading to tumor cell death. It also forms DNA adducts with other cellular components such as RNA, proteins, and lipids (Arora et al., 2022). Cisplatin's wide range of antitumor activity is limited by its side effects, including ototoxicity, nephrotoxicity, hepatotoxicity, and neurotoxicity (Xu and Gewirtz, 2022). Cisplatin has also been reported to cause optic nerve changes, such as neuritis and ischemia (Chiang et al., 2022).

Oxidative stress (OS) is the discrepancy among free radicals, i.e., reactive oxygen, nitrogen species, and advanced glycated-end products (AGEs), which are generally produced by several pathways and the intrinsic antioxidant scavenging system. Once free radicals rise, they overwhelm intrinsic antioxidant defenses (Nebbioso et al., 2022). Oxidative stress can damage lipids, carbohydrates, proteins, enzymes, and nuclear and mitochondrial DNA, as a consequence of which cell death is induced. OS is associated with several neurodegenerative and ocular disorders (Domènech and Marfany, 2020). The endoplasmic reticulum (ER) is well-known as the protein-manufacturing organelle within the cell (Zhang et al., 2014). It is engaged in the biosynthesis, post-translational modification, folding, and trafficking of proteins (Zhang et al., 2014). Unfolded protein response (UPR) is a complex surveillance system within the ER that detects misfolded proteins. It is a series of signaling cascades that are initiated by ER stress (Zhang et al., 2014). Upon insult to the ER, the UPR activates three ER transmembrane enzymes, including activating transcription factor 6 (ATF6), protein kinase R-like endoplasmic reticulum kinase (PERK), and inositol-requiring enzyme 1 (IRE1). Glucose-regulated protein 78 (GRP78), also named as binding immunoglobulin protein (BiP), is an ER-resident protein chaperone. UPR signaling is initiated via dynamic interaction between GRP78 and three enzymes of the ER (Gorbatyuk et al., 2020). When the intensity and extent of ER stress exceed the UPR's capacity to restore homeostasis, the apoptotic cascade is activated (Zhang et al., 2014). C/EBP homologous protein (CHOP) expression is stimulated by UPR, mainly via the PERK pathway. CHOP activation is recognized as a core prompt of ER stress-induced apoptosis (Yang et al., 2017). IRE1 has the ability to cleave and activate X-box-binding protein 1 (XBP1) as well as potentiate the pro-apoptotic c-Jun N-terminal (JNK) (Siwecka et al., 2021).

Autophagy is a degradation process that eradicates injured organelles and unfolds proteins to preserve cellular homeostasis (Lin and Xu, 2019). The autophagosome is the hallmark of autophagy. It is a double-membrane vesicle that engulfs distorted cytosolic components, organelles, proteins, and cellular debris and then consequently merges with the lysosome (Lin and Xu, 2019). The autophagic cascade defines the whole dynamic of the autophagy process, from autophagosome creation to the degradation and recycling of its contents (Lin and Xu, 2019). Beclin 1 regulates autophagy and membrane trafficking, which are crucial for many physiological and pathological processes (Kang et al., 2011). Microtubule-associated protein 1 light chain 3 (MAP1L C3/LC3) is a homolog of yeast autophagy-related protein Atg8 that is commonly assessed as an indicator of the phagophore or autophagosome. Sequestosome 1 (SQSTM1/p62) is considered one of the most important autophagy receptors that binds ubiquitinated proteins to LC3 (Klionsky et al., 2021). p62 is degraded by autophagy, and its impairment leads to the accumulation of p62 within cells (Zhang and Costa, 2021). Thus, the current study was designed to investigate the mitigative outcome of tempol against cisplatin-induced optic neuropathy regarding oxidative burst, ER stress, and the autophagy signaling pathway.

## 2. Materials and methods

### 2.1. Experimental animals

In this study, forty-eight adult male albino Wistar rats 200–250 gm and 6–8 weeks old were obtained from the animal house of the faculty of Medicine Zagazig University. They were kept in metal clean and well-ventilated cages with a 12 h light/12 h dark cycle at room temperature. The rats were fed on a standard rat-chewable diet and provided with clean water *ad libitum*. Before any experimental procedures, rats were left for a period of acclimation for 7 days. All the experimental procedures were carried out according to the guidelines for the care and use of laboratory animals and were approved by the Institutional Animal Care and Use Committee (IACUC) of Zagazig University with a reference number (ZU-IACUC/3/F/205/2019).

### 2.2. Chemicals

Cisplatin was purchased as commercially available vials of cisplatin (Mylan Pharmaceuticals, France). Tempol powder (4-Hydroxy-TEMPO) was obtained from Sigma-Aldrich, CAS-No. 2226-96-2). A saline [0.9% sodium chloride (NaCl)] solution was bought from El-Nasr Pharmaceutical Chemicals Company (ADWIC), Egypt.

### 2.3. Experimental design

Rats were randomly assigned into four equal groups of 12 rats each. The control group rats were injected intraperitoneally daily with 0.5 mL of normal saline for 14 days. The tempol-treated (TEM) group rats were injected intraperitoneally with 0.5 mL of tempol (100 mg/kg/day) reconstituted in saline for 14 days (Ewees et al., 2019). The cisplatin-treated (CIS) group rats were subjected to a single intraperitoneal injection of cisplatin (7 mg/kg/day) on day 8 of the study (Gul Baykalir et al., 2018). Cisplatin and tempol-treated (CIS + TEM) group rats were injected intraperitoneally with 100 mg/kg/day of tempol for 14 days, and a single intraperitoneal injection of cisplatin (7 mg/kg) on day 8 of the study (Gul Baykalir et al., 2018; Ewees et al., 2019).

### 2.4. Specimen collection

24 h after the end of the experiment, the rats were anesthetized using an intraperitoneal injection of thiopental (50 mg/kg). Afterward, the optic nerves were extracted. To dissect the optic nerve, a craniotomy was performed, followed by gentle retraction of the brain and then cutting off the nerve behind the orbit and just before the optic chiasma (Bolton and Butt, 2005). The right optic nerves were processed for histological examination; they were equally divided as follows: six samples were treated for histopathological examination, and the others were processed for electron microscopy. The left optic nerves were equally divided as follows: six samples were prepared for biochemical (tissue homogenates) analysis, and the others were prepared for reverse transcriptase polymerase chain reaction (RT-PCR) analysis.

### 2.5. Oxidant/antioxidant activity

The optic nerve homogenates were used for the oxidant and antioxidant assays, malondialdehyde (MDA), superoxide dismutase (SOD), and glutathione peroxidase (GPx). MDA was detected as an end product of lipid peroxidation, according to Ohkawa et al. (1979) and Ertik et al. (2023). SOD activity was measured as previously mentioned by Kizir et al. (2023). GPx enzymatic activity was performed as per the method used by Ertik et al. (2023).

### 2.6. RT-qPCR for gene expression

The extraction procedures of the RNA from the optic nerve were carried out with Qiazol (Qiagen; Hilden, Germany), following the supplier's instructions, and the RNA concentration was measured and adjusted to 500 ng/μL of RNAse free water using a NanoDrop^®^ ND−1000 spectrophotometer (NanoDrop Technologies, USA) according to Abdullah et al. (2022). The synthesis of the complementary DNA strands (cDNA) was carried out with 500 ng of total RNA with a high-capacity cDNA reverse transcription kit (Applied Biosystems™, USA) as per the manufacturer procedures with a 20 μL reaction volume. The gene expression study was performed using a RotorGene Q 2 plex thermal cycler real-time system (Qiagen, Germany) as per Khamis et al. (2019, 2021) and Khamis et al. (2022), in a 20 μL reaction volume [10 μL sybergreen TOPreal™ qPCR 2 × PreMIX (Enzynomics, Korea), 1 μL forward and reverse primers (Sangon Biotech, China) (Table 1), 1 μL of prediluted cDNA 1:25, and 7 μL RNAse free water (Enzynomics, Korea)] following the method described by Khamis et al. (2019, 2021) and Khamis et al. (2022). The relative expression of the target genes was calculated using the method developed by Schmittgen and Livak (2008), the cycle threshold (CT) of the target gene was normalized using the stably expressed internal control reference gene *Gapdh* to calculate Δct value, and then, the ΔΔCT was calculated as sample ΔCT—control average ΔCT, and the fold change of the target gene was estimated using the 2^−ΔΔCT^ equation.

**Table 1:** Primers used in PCR.

**Gene**	**Forward primer sequence (5^′^to 3^′^)**	**Reverse primer sequence (5^′^to 3^′^)**	**Product size**	**Accession no**.
ATF6	AAGTGAAGAACCATTACTTTATATC	TTTCTGCTGGCTATTTGT	157	NM_001107196.1
Beclin 1	GAATGGAGGGGTCTAAGGCG	CTTCCTCCTGGCTCTCTCCT	180	NM_001034117.1
BiP	AACCAAGGATGCTGGCACTA	ATGACCCGCTGATCAAAGTC	240	NM_013083.2
CHOP	CACAAGCACCTCCCAAAG	CCTGCTCCTTCTCCTTCAT	158	NM_001109986.1
Gapdh	GGCACAGTCAAGGCTGAGAATG	ATGGTGGTGAAGACGCCAGTA	143	NM_017008.4
JNK	AGTGTAGAGTGGATGCATGA	ATGTGCTTCCTGTGGTTTAC	182	NM_053829.2
LC3	GAAATGGTCACCCCACGAGT	ACACAGTTTTCCCATGCCCA	147	NM_012823.2
NGF	AGCGCATCGCTCTCCTT	TGGCCAGGATAGAAAGCTGC	139	NM_001277055.1
p62	GTGGAACCCCAGTAAGAGGC	TGAATACCAGCTGTCCGAGC	115	NM_181550.2
XBP1	TTACGAGAGAAAACTCATGGGC	GGGTCCAACTTGTCCAGAATGC	289	NM_001004210.2

### 2.7. Histopathological analysis

The fixation of the optic nerve samples was carried out using neutral-buffered formalin 10%. The tissue dehydration was then carried out in an ascending concentration of ethyl alcohol and finally cleared in xylene, following which the tissues were impregnated and embedded in paraffin wax. The paraffin blocks were sectioned (5 μm) and stained with hematoxylin and eosin (HandE) (Suvarna et al., 2019). The HandE-stained sections were inspected using a light microscope (Olympus BX41^®^ microscope).

### 2.8. Immunohistochemical staining

Immunohistochemical analysis was conducted to assess the protein expression of autophagy markers (LC3-II and p62). The optic nerve paraffin blocks were sliced into 4 μm sections, deparaffinized in xylene, and rehydrated with descending ethyl alcohol concentrations. To inhibit tissue endogenous peroxidase, the sections were incubated with 3% hydrogen peroxide for 5 min. For antigen retrieval, the sections were incubated for 15 min in 400 ml of 0.01 M citrate buffer (pH 6.0) and then rinsed with phosphate-buffered saline (PBS). To block the non-specific antigen, the sections were incubated in 10% goat serum (#50197Z, Thermo Fisher Scientific). The sections were then incubated with primary antibodies of LC3-II rabbit monoclonal antibodies (mAb) (#12741, cell signaling technology) with a dilution of 1:500 and SQSTM1/p62 rabbit mAb (#23214, cell signaling technology) with a dilution of 1:250 at 4°C overnight. Thereafter, the sections were washed twice with PBS for 5 min, followed by incubation with the specific biotinylated secondary antibody for 30 min, and then, the avidin–biotin complex (Vectastain^®^ ABC-peroxidase kit, Vector Laboratories, Burlingame, CA) was added and incubated for another 30 min. Finally, the chromogen 3,3′-diaminobenzidine tetrahydrochloride hydrate (DAB HCl) (#D5637, Sigma-Aldrich) was added to visualize the immunoreaction. Eventually, the sections were counterstained with Mayer's hematoxylin, dehydrated, and mounted on Canada balsam (Taylor, 2014). The stained section was inspected using a light microscope (Leica Microsystems, Schweiz, AG, Heerbrugg, CH-9435, Switzerland) in the Image Analysis Unit of the Anatomy Department, Faculty of Medicine, Zagazig University.

### 2.9. Electron microscopic studies

The fixation of the optic nerve samples was performed with 2.5% glutaraldehyde for 24 h at 4°C after cutting them into thin strips 1 mm^3^. The fixed optic nerves were then washed with PBS, followed by post-fixation in a buffered solution of 1% osmium tetroxide for 60–90 min. The tissues were subsequently washed in a 1% osmium tetroxide buffered solution. For the tissue rehydration, optic nerves were passed serially in an increasing concentration of alcohol of 70, 90, 95, and 100%; then, the alcohol was cleared by immersion in propylene oxide twice and embedded in epoxy resin. Semi-thin sections (0.5–1 μm) were obtained by using an LKB ultramicrotome with glass knives, mounted on glass slides, and stained with toluidine blue. For the selection of the suitable area for the electron microscopy study, the toluidine blue-stained section was examined using the light microscope. Ultrathin sections (80 nm) were obtained from the preselected areas, mounted on copper grids, and contrasted with uranyl acetate and lead citrate (Woods and Stirling, 2019). The sections were examined using a Talos L120C G2 transmission electron microscope (ThermoFisher, Europe), Electron Microscope Unit, Faculty of Agriculture, Damietta University.

### 2.10. Morphometric analysis

Morphometric analysis was conducted to evaluate the immunohistochemical results. The LC3 and p62 positive area percentages were measured by FIJI/ImageJ^®^ software (1.51 n, NIH, USA). The analysis was performed using 3 sections per rat at a 400× magnification (6 rats per group) in all experimental groups. Concisely, using Image J software, the deconvoluted DAB images were converted to grayscale (8-bit color) to reduce background color and maximize signal separation. Based on the intensity of signals, the threshold was adjusted for DAB detection. All threshold parameters were maintained throughout all images (Schindelin et al., 2012).

### 2.11. Statistical analysis

Before group comparisons, all datasets were checked for normality. Consequently, parametric analyses were performed, and the values taken were the mean of six samples per group ± standard error of the mean (SEM). The statistical comparisons between the mean of the different experimental groups were performed through a one-way analysis of variance (ANOVA). The *post-hoc* analysis was performed by Tukey's tests for multiple comparisons. The statistical analysis was conducted using GraphPad Prism^®^, Version 9.2 (GraphPad Software Inc., San Diego, California, USA). The results were considered statistically significant when the *p*-value was <0.05.

## 3. Results

### 3.1. Effect of tempol administration on the oxidative stress markers in cisplatin-induced optic neuropathy

**Figure 1:**
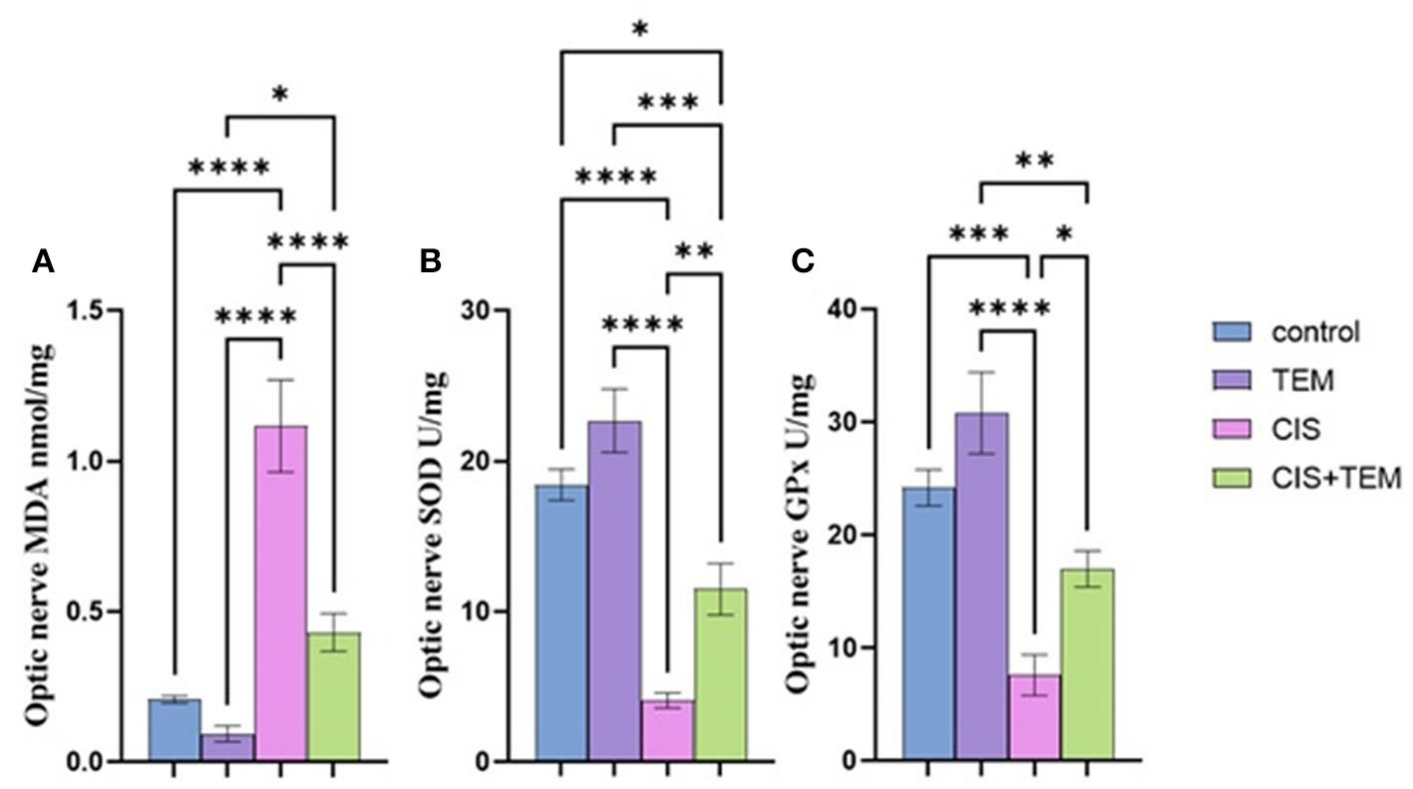
Effect of tempol administration on the oxidative stress markers in cisplatin-induced optic neuropathy **(A–C)**. **(A)** The mean value of MDA nmol/mg of the different experimental groups. **(B)** The mean value of SOD U/mg of the different experimental groups. **(C)** The mean value of GPx U/mg of the different experimental groups. Values are the mean of six rats per group ± S.E.M. *p < 0.05, **p < 0.01, ***p < 0.001, and ****p < 0.0001.

Cisplatin significantly (*p* < 0.0001) increased the lipid peroxidation marker MDA and reduced the antioxidant markers, namely superoxide dismutase (SOD) and glutathione peroxidase (GPx), in comparison with the control and tempol-treated groups (Figures 1A–C). In contrast, pre- and co-treatment with tempol significantly reduced the lipid peroxidation marker MDA (*p* < 0.05) and significantly increased the antioxidant markers SOD (*p* < 0.01) and GPx (*p* < 0.001) compared with the cisplatin-treated group (Figures 1A–C). However, the TEM + CIS group revealed a significant increase in the lipid peroxidation marker MDA (*p* < 0.05) (Figure 1A) and a decrease in GPx activity (*p* < 0.01) (Figure 1C) compared to the TEM group. Furthermore, the results showed a significant decrease in SOD activity in the TEM + CIS group compared to the control (*p* < 0.05) and TEM group (*p* < 0.001) (Figure 1B).

### 3.2. Effect of tempol administration on the mRNA expression of the endoplasmic reticulum in cisplatin-induced optic neuropathy

**Figure 2:**
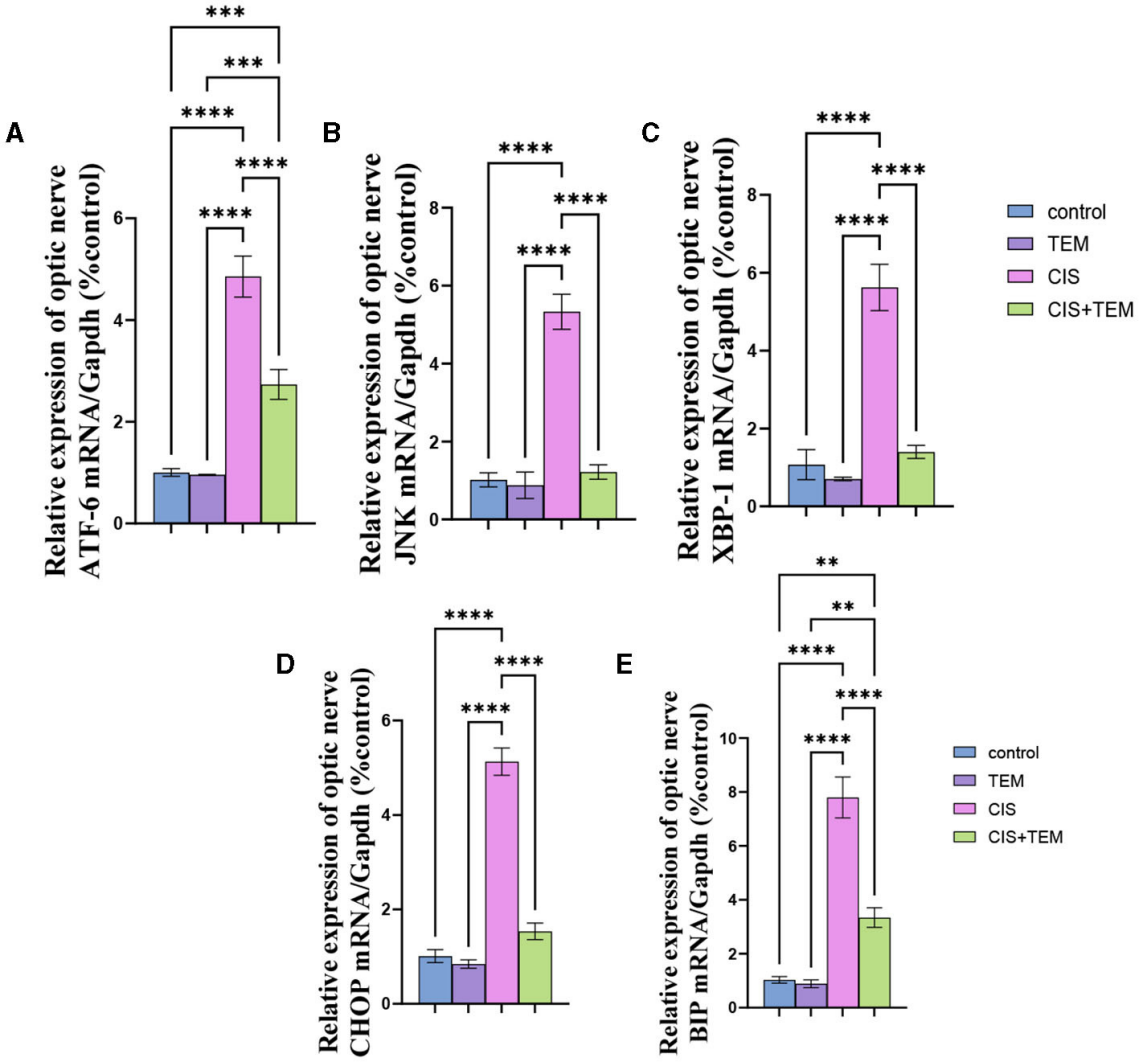
Effect of tempol administration on the mRNA expression of the endoplasmic reticulum in cisplatin-induced optic neuropathy **(A–E)**. Relative mRNA expression of optic nerve ER stress markers—**(A)** ATF-6/Gapdh; **(B)** JNK/Gapdh; **(C)** XBP-1/Gapdh; **(D)** CHOP/Gapdh; and **(E)** BIP/Gapdh. Values are the mean of six rats per group ± S.E.M. **p < 0.01, ***p < 0.001, and ****p < 0.0001.

The cisplatin group showed a significant (*p* < 0.0001) upregulation in the relative mRNA expression of endoplasmic reticulum stress markers in the optic nerve—*ATF6, BiP, XBP1, CHOP*, and *JNK*—compared with the control and tempol-treated groups (Figures 2A–E). On the contrary, the Cis + TEM group revealed a significant (*p* < 0.0001) downregulation in the relative mRNA expression of the aforementioned ER stress markers compared with the CIS group (Figures 2A–E). Additionally, the TEM + CIS group showed a significant (*p* < 0.001) upregulation in the mRNA expression of *ATF-6* and *BiP* compared with the control and TEM groups (Figure 2A).

### 3.3. Effect of tempol administration on the expression of Beclin-1 and LC3 in cisplatin-induced optic neuropathy

**Figure 3:**
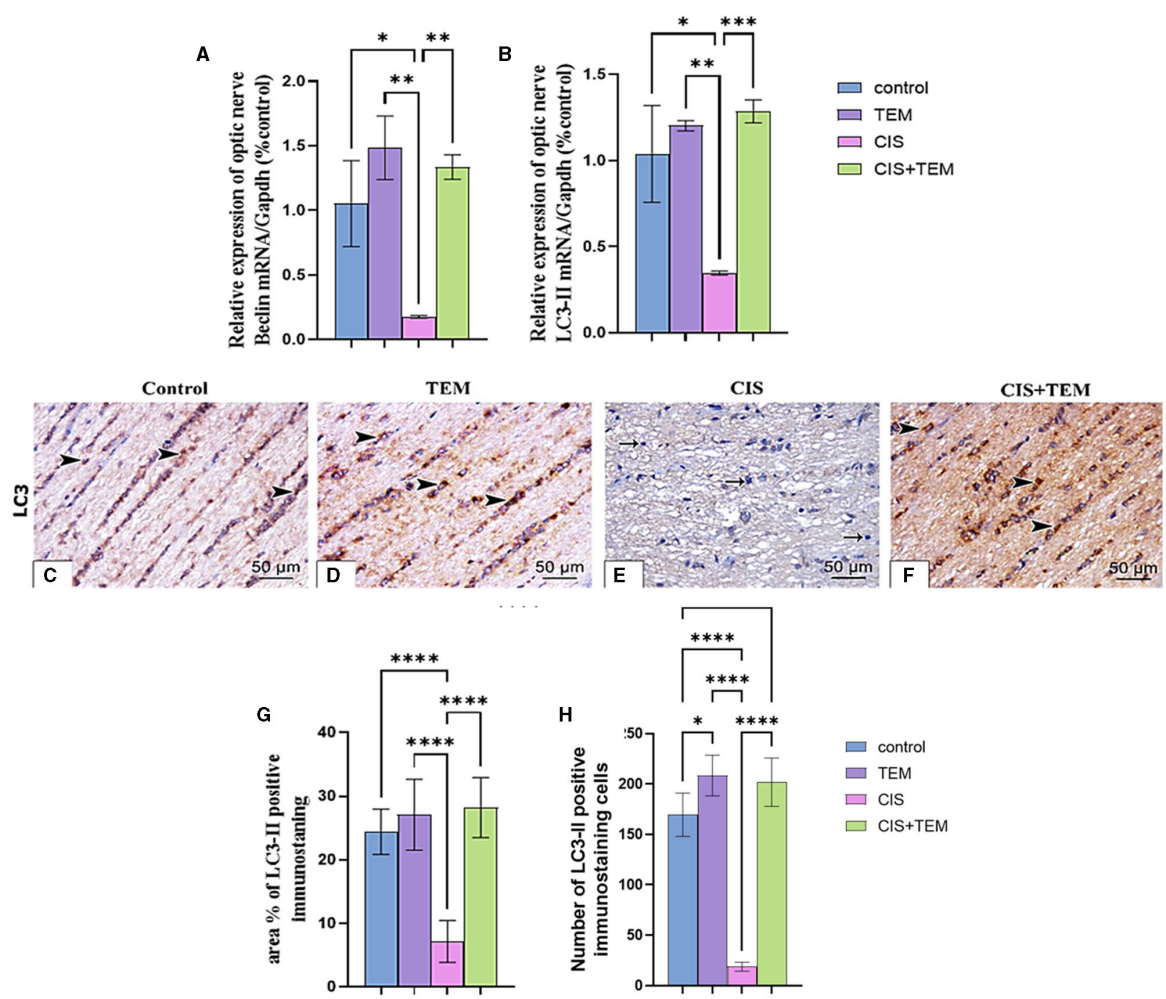
Effect of tempol administration on the expression of Beclin-1 and LC3 II in cisplatin-induced optic neuropathy **(A–G). (A, B)** Relative mRNA expression of optic nerve Beclin-1/Gapdh and LC3 II/Gapdh, respectively. **(C–F)** Representative photomicrographs showing the estimation of immunohistochemical staining of LC3 II in optic nerves among the different experimental groups. **(C)** Control group; **(D)** TEM group; **(E)** CIS group; and **(F)** CIS. The positive immune expression can be observed as brown discoloration (arrowheads), whereas arrows indicate weak immune expression. Scale bar: 50 μm × 400. **(G)** sArea % of LC3-II positive immunostaining in different experimental groups. **(H)** The number of LC3-II positive immunostained cells in different experimental groups. Values are the mean of six rats per group ± S.E.M. *p < 0.05, **p < 0.01, ***p < 0.001, and ****p < 0.0001.

Cisplatin administration induced significant downregulation of LC3 mRNA, immunohistochemical protein expression, and Beclin-1 mRNA expression compared with the control (*p* < 0.05) and tempol-treated groups (*p* < 0.01) (Figures 3A–H). In contrast, administration of tempol induced significant upregulation of LC3 mRNA, immunohistochemical protein expression (*p* < 0.001), and Beclin-1 mRNA (*p* < 0.01) expression compared with the cisplatin-treated group (Figures 3A–H). Interestingly, the aforementioned autophagy markers in CIS + TEM revealed a non-significant difference in the control and TEM groups (Figures 3A–H).

### 3.4. Effect of tempol administration on the expression of p62 in cisplatin-induced optic neuropathy

**Figure 4:**
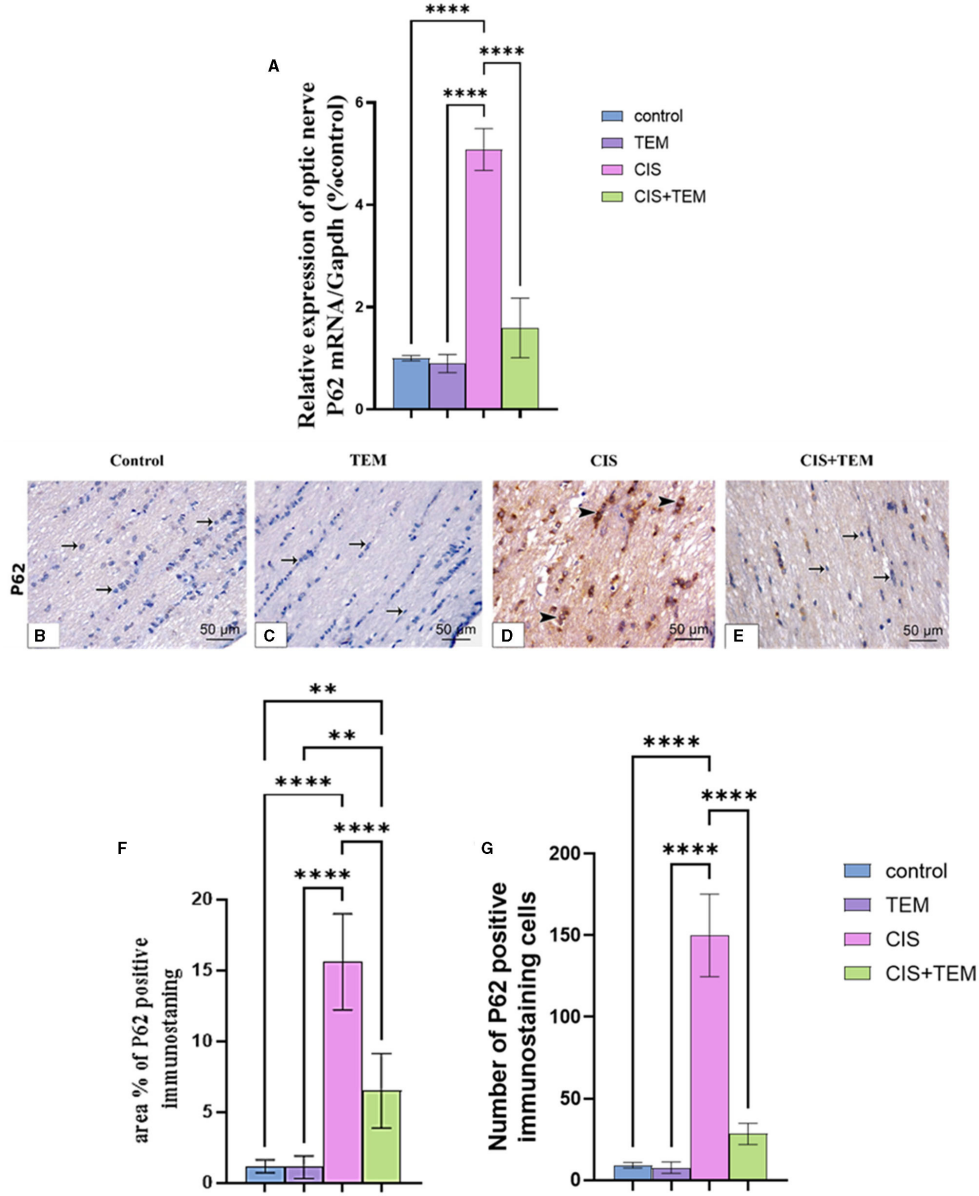
Effect of tempol administration on the expression of p62 in cisplatin-induced optic neuropathy **(A–G)**. **(A)** Relative mRNA expression of optic nerve p62/Gapdh. **(B–G)** Representative photomicrographs showing the estimation of immunohistochemical staining of p62 in optic nerves among the different experimental groups. **(B)** Control group; **(C)** TEM group; **(D)** CIS group; and **(E)** CIS + TEM group. The positive immune expression can be observed as brown discoloration (arrowheads), whereas arrows indicate weak immune expression. Scale bars: 50 μm × 400. **(F)** Area % of p62 positive immunostaining in different experimental groups. **(G)** Number of p62-positive immunostained cells in different experimental groups. Values are the mean of six rats per group ± S.E.M. **p < 0.01 and ****p < 0.0001.

The cisplatin-treated group exhibited a significant (*p* < 0.0001) upregulation of p62 mRNA and immunohistochemical protein expression compared to the control and TEM-treated groups (Figures 4A–G). However, the CIS + TEM group elicited a significant (*p* < 0.0001) downregulation of p62 mRNA and immunohistochemical protein expression compared to the CIS group (Figures 4A–G). However, p62 immunohistochemical protein expression in the TEM + CIS group still revealed a significant (*p* < 0.01) difference from the control and TEM groups (Figures 4A–G).

### 3.5. Effect of tempol administration on the mRNA expression of nerve growth factor and histopathological change in cisplatin-induced optic neuropathy

**Figure 5:**
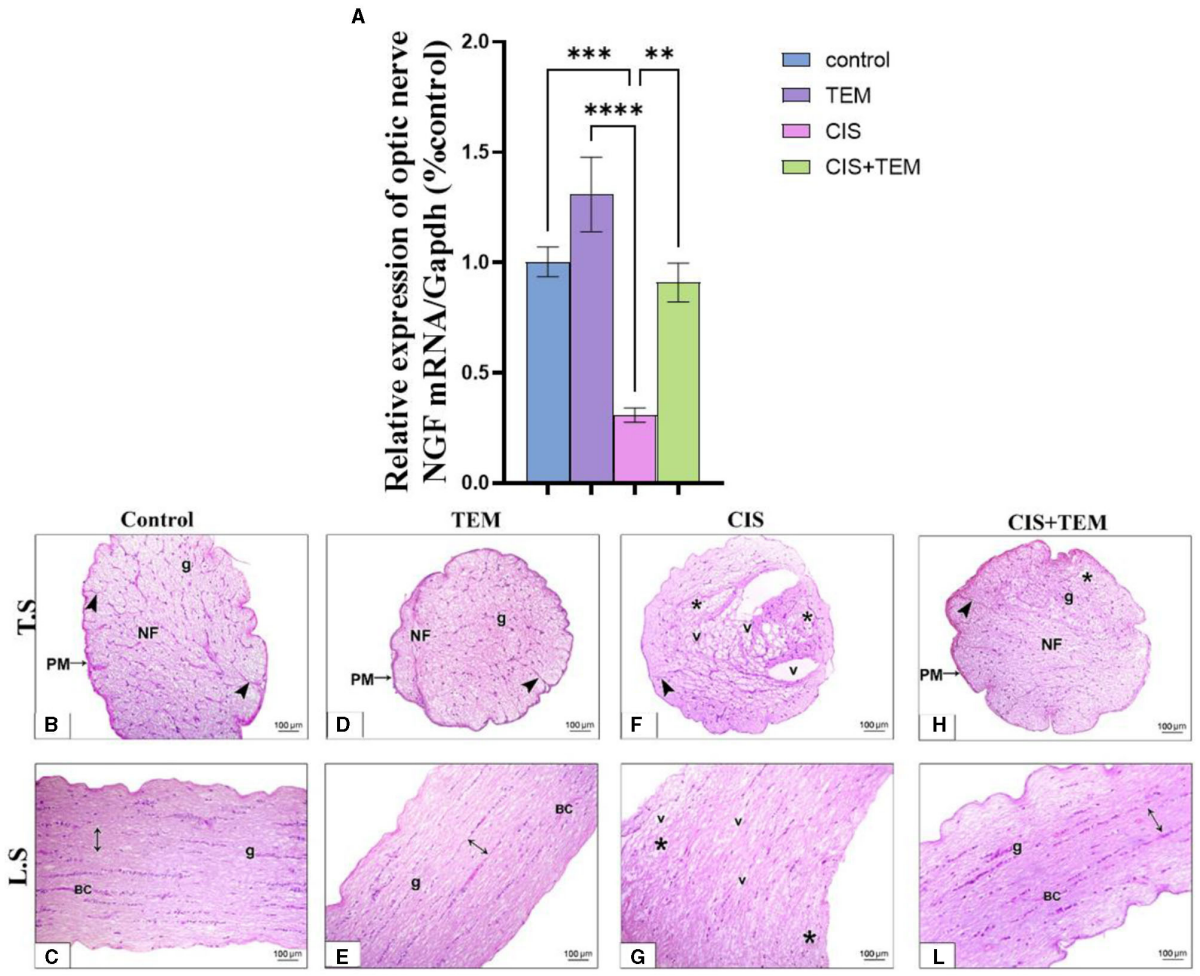
Effect of tempol administration on the mRNA expression of nerve growth factor and histopathological change in cisplatin-induced optic neuropathy **(A–L)**. **(A)** The bar chart exhibits a relative mRNA expression of optic nerve NGF-1/Gapdh. **(B–L)** Representative photomicrographs of transverse and longitudinal sections of optic nerve among the various experimental groups. **(B, C)** and **(D, E)** Control and TEM groups, respectively; **(B, D)** show a normal structure of the optic nerve where the pia mater (PM) ensheathes the closely packed nerve axons in transverse sections of both groups, which are segregated into fascicles (NF) by thin branching connective tissue trabeculae (arrowheads) comprising occasional blood capillaries. The nuclei of the glial cells (g) are dispersed between the axons. **(C, E)** Show parallel nerve fibers (double-sided arrow) in longitudinal sections of both groups with occasional blood capillaries (BC) in the nerve trabecula, in addition to several series of glial cells (g). **(F, G)** CIS groups in both transverse and longitudinal sections, respectively, show disrupted nerve structure. The nerve fascicles are disorganized (*) with less organized connective tissue trabeculae (arrowhead) in between. Additionally, vacuoles (v) of variable sizes are observed in the nerve tissue. **(H, L)** The CIS + TEM group shows nearly preserved optic nerves. **(H)** Shows the pia mater (PM) that ensheathes the closely packed nerve axons in the transverse section, which are segregated into fascicles (NF) by thin branching connective tissue trabeculae (arrowhead) comprising occasional blood capillaries. However, few disorganized fibers are observed (*). The nuclei of the glial cells (g) are dispersed between the axons. **(L)** Shows the parallel nerve fibers (double-sided arrow) in the longitudinal section with occasional blood capillaries in the nerve trabecula, in addition to several series of glial cells (g). Scale bars: 100 μm × 200. ^**^p < 0.01, ^***^p < 0.001, and ^****^p < 0.0001.

Cisplatin significantly downregulated the mRNA expression of nerve growth factor (NGF) in comparison with the control (*p* < 0.001) and TEM-treated groups (*p* < 0.0001) (Figure 5A). However, pre- and co-treatment with tempol significantly (*p* < 0.01) upregulated the mRNA expression of *NGF* in comparison with the CIS-treated group and still revealed a significant difference from the control and TEM groups (Figure 5A). A microscopic examination of HandE-stained transverse sections (TS) of the optic nerve tissues in the different experimental groups is illustrated in Figures 5B–L). Both the control and TEM-treated groups revealed normal histological appearance, where the pia mater ensheathed the closely packed nerve axons, which were segregated into fascicles by thin branching connective tissue trabeculae comprising occasional blood capillaries. The nuclei of the glial cells were also dispersed between the axons (Figures 5B, D). Whereas, in the CIS-treated group, there was disorganization of the nerve fascicles, as well as less organized connective tissue trabeculae in between. Additionally, vacuoles of variable sizes were observed (Figure 5F). However, in the cisplatin group pre- and co-treated with tempol, the nerve architecture was nearly preserved, except for a few disorganized fibers. The pia mater ensheathed the closely packed nerve axons, which were segregated into fascicles by thin branching connective tissue trabeculae comprising occasional blood capillaries. The nuclei of glial cells were also dispersed between the axons (Figure 5H). The microscopic examination of HandE-stained longitudinal sections (LS) of the optic nerve tissues in the different experimental groups is illustrated in Figures 5C–I). The control and TEM-treated groups revealed a normal histological appearance since the nerve fibers were parallel with occasional blood capillaries in the nerve trabecula, in addition to several series of glial cells (Figures 5C, E). Meanwhile, in the CIS-treated group, there was disorganization of the nerve fibers with vacuolations in between (Figure 5G). On the contrary, in the cisplatin group pre- and co-treated with tempol, the nerve architecture was nearly preserved. The nerve fibers were parallel with occasional blood capillaries in the nerve trabecula, in addition to several series of glial cells (Figure 5L).

### 3.6. Effect of tempol administration on the optic nerve ultrastructure in cisplatin-induced optic neuropathy

**Figure 6:**
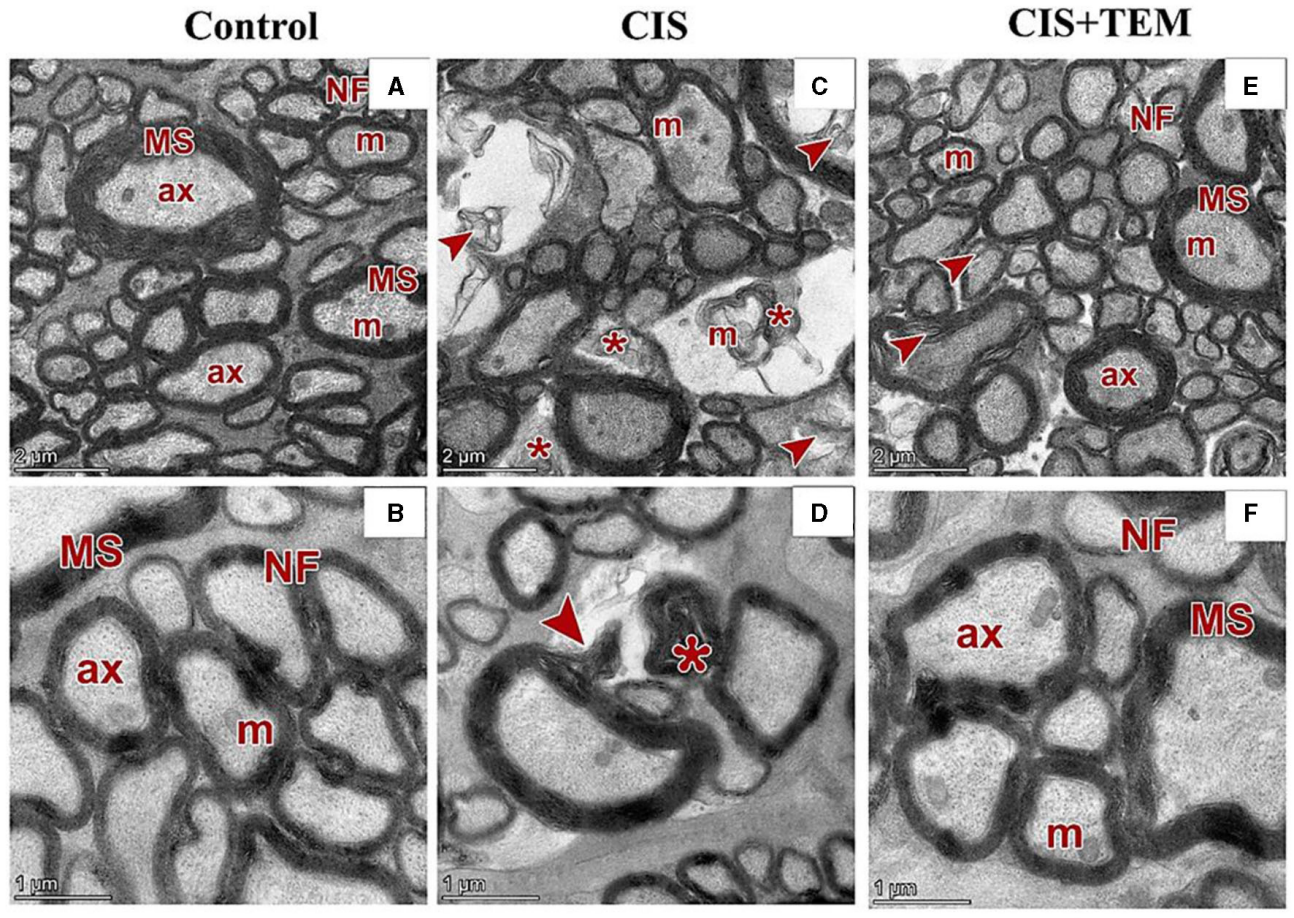
Effect of tempol administration on the ultrastructures (astrocytes and oligodendrocytes) of the optic nerve in cisplatin-induced optic neuropathy. **(A–F)** Representative electron micrographs of ultrathin sections of optic nerves among the various experimental groups. **(A, B)** The control group shows the nerve fibers (NF) that are packed together and have variable sizes. The axons are encircled by compact myelin sheaths (MS) of variable thickness based on their size and exhibit pale axoplasms (ax) with varying degrees of electron-lucent structures—microtubules and neurofilaments—in addition to occasional mitochondria (m). **(C, D)** The CIS group shows distorted nerve fibers (arrowheads). They display deformed myelination with disorganized myelin sheets as well as myelin whorl (*) formation. There are also swollen mitochondria (m) in some axons. **(E, F)** The CIS+TEM group shows the nerve fibers (NF) that are packed together and have variable sizes. The axons are encircled by myelin sheaths (MS) of variable thickness based on their size and exhibit pale axoplasms (ax) with varying degrees of electron-lucent structures—microtubules and neurofilaments—in addition to occasional mitochondria (m). Even so, few distorted nerve fibers (arrowheads) are present among the normal ones. Scale bars in **(A, C, E)**: 2 μm × 2,600; scale bars in **(B, D, F)**: 1 μm × 5,300.

**Figure 7:**
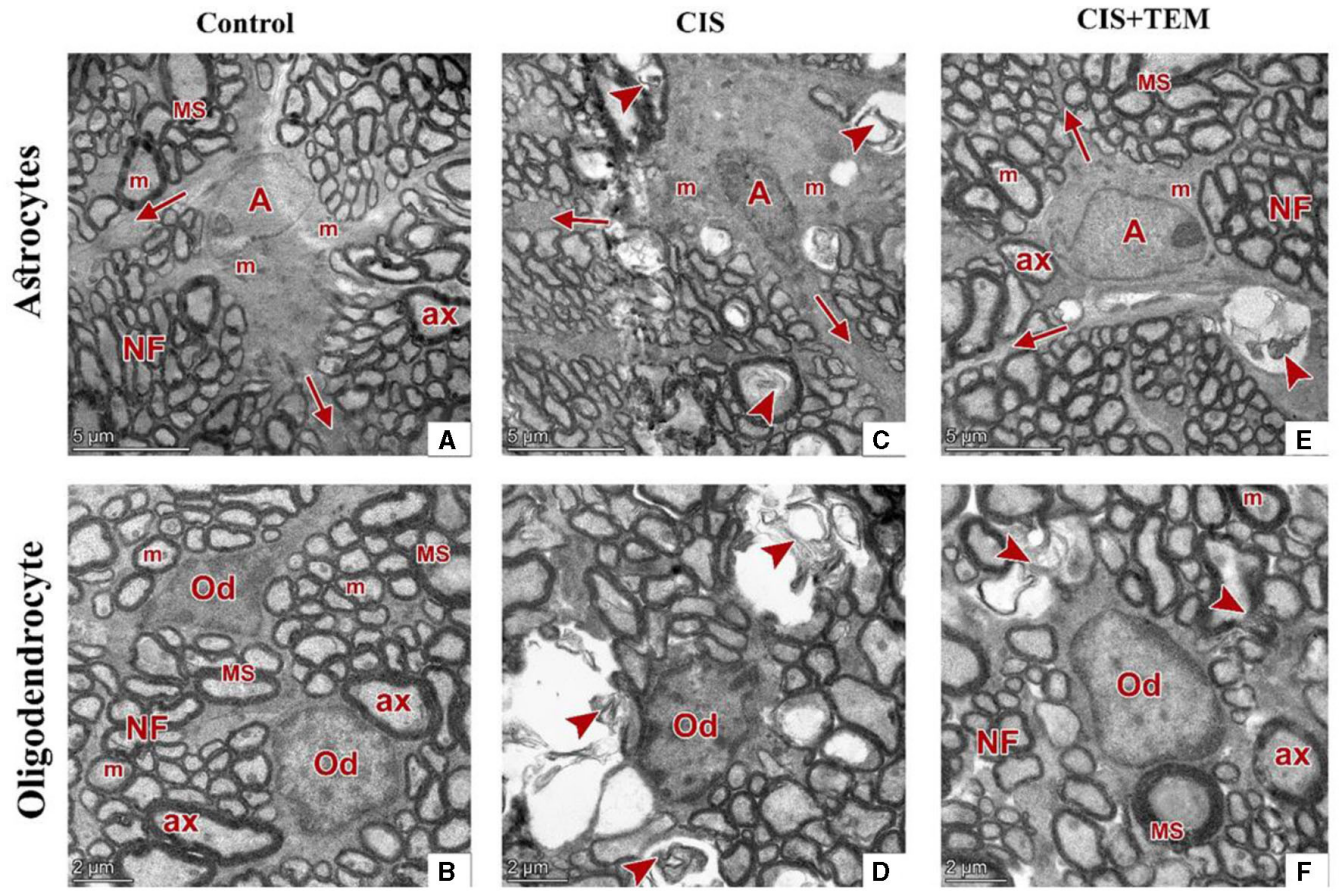
Effect of tempol administration on the ultrastructure (nerve cells) of the optic nerve in cisplatin-induced optic neuropathy. **(A–F)** Representative electron micrographs of ultrathin sections of optic nerves among the various experimental groups. **(A, B)** The control group shows an astrocyte **(A)** with several processes (arrows) separating the axons. It has large euchromatic nuclei with thin peripheral heterochromatin and cytoplasm that comprise mainly mitochondria (m). The oligodendrocytes (Od) appear between nerve fibers. They are moderately electron-dense with slightly ovoid nuclei comprising peripherally clumped chromatin. The nerve fibers (NF) are arranged in fascicles and separated by astrocytic processes. The axons were encircled by compact myelin sheaths (MS) of variable thickness and exhibited pale axoplasms (ax) with varying degrees of electron-lucent structures in addition to occasional mitochondria (m). **(C, D)** The CIS group shows an astrocyte **(A)** with an irregular nucleus, which is more electron-dense than that in the control group, and a cytoplasm that comprises swollen mitochondria (m). The oligodendrocyte (Od) appears between distorted nerve fibers (arrowheads). It has an irregular nucleus that is more electron-dense than that of the control group. The distorted nerve fibers are arranged in fascicles and separated by astrocytic processes (arrows). **(E, F)** The CIS+TEM group shows an astrocyte **(A)** with several processes (arrows) separating the axons. It has a large euchromatic nucleus with thin peripheral heterochromatin and cytoplasm that comprises mainly mitochondria (m). The oligodendrocyte (Od) appears between nerve fibers. It is moderately electron-dense with a slightly ovoid nucleus comprising peripherally clumped chromatin. The nerve fibers (NF) are arranged in fascicles and separated by astrocytic processes. However, few distorted nerve fibers (arrowhead) are present among the normal ones. The axons are encircled by compact myelin sheaths (MS) of variable thickness and exhibit pale axoplasms (ax) with varying degrees of electron-lucent structures in addition to occasional mitochondria (m). Scale bars **(A, C, E)**: 5 μm × 1,300; scale bars **(B, D, F)**: 2 μm × 1,600.

The control and TEM-treated groups revealed the same ultrastructural findings of the optic nerve; therefore, the control group was selected for interpretation of the results. Ultrastructural examination of the optic nerves in the control group revealed normal architecture. Optic nerve fibers were packed together and had variable sizes. They were arranged in fascicles and separated by astrocytic processes. The axons were encircled by compact myelin sheaths of variable thickness based on their size and exhibited pale axoplasms with varying degrees of electron-lucent structures—microtubules and neurofilaments—in addition to occasional mitochondria (Figures 6A, B). Astrocytes had large euchromatic nuclei with thin peripheral heterochromatin, and their cytoplasm comprised mainly mitochondria. Additionally, they possessed several long processes separating the axons (Figure 7A). Oligodendrocytes were observed between the axons. They seemed moderately electron-dense, with slightly ovoid nuclei comprising peripherally clumped chromatin (Figure 7B).

Ultrastructural examination of the optic nerves in the CIS-treated group revealed disrupted architecture. Optic nerve fibers were distorted. They displayed deformed myelination with disorganized myelin sheets as well as myelin whorl formation. There were also swollen mitochondria in some axons (Figures 6C, D). Astrocytes had irregular nuclei that were more electron-dense than those in the control group, and their cytoplasm comprised swollen mitochondria (Figure 7C). Oligodendrocytes had irregular nuclei that were more electron-dense than those in the control group (Figure 7D). Ultrastructural examination of the optic nerves in the CIS group pre- and co-treated with tempol revealed comparatively preserved architecture. Optic nerve fibers were packed together and had variable sizes. They were arranged in fascicles and separated by astrocytic processes. The axons were encircled by compact myelin sheaths of variable thickness based on their size and exhibited pale axoplasms with varying degrees of electron-lucent structures—microtubules and neurofilaments—in addition to occasional mitochondria. Even so, few distorted nerve fibers were present among the normal ones (Figures 6E, F). Astrocytes had large euchromatic nuclei with thin peripheral heterochromatin, and their cytoplasm comprised mainly mitochondria. Additionally, they possessed several long processes separating the axons (Figure 7E). Oligodendrocytes were observed between the axons. They seemed moderately electron-dense, with slightly ovoid nuclei comprising peripherally clumped chromatin (Figure 7F).

## 4. Discussion

Cisplatin has been found to have an affinity for neuronal structures in the central nervous system (Cankara et al., 2021). The eye is protected from peripheral circulation by the blood-ocular barriers (BOBs) (Kaur et al., 2008; Rizzolo et al., 2011). The BOBs are comprised of two barriers, including the blood-aqueous barrier and the blood-retinal barrier (BRB), which is one of the blood-nerve barriers (Cunha-Vaz, 1979, 2010). Rats that received cisplatin-based chemotherapy had arterial occlusion and endothelial damage. In cisplatin-treated rats, reduced nerve blood flow, decreased number of vasa nervorum, and endothelial apoptosis have been reported. Recently, the lower density and detachment of nerve pericytes have been found in rats with cisplatin neuropathy. These abnormalities impair the blood-nerve barrier (BNB) (Kirchmair et al., 2005; Li et al., 2006; Dieckmann et al., 2011; Jindatip et al., 2019) and eventually impact its permeability to substances. The optic nerve serves as an ideal model for studying axonal physiology and pathology in the central nervous system because of its surgical accessibility and well-defined anatomy. Therefore, the optic nerve has been used in several experimental models to explore the general mechanisms of neuronal axonal degeneration (Koch and Lingor, 2016). Therefore, the optic nerve was selected as a model of cisplatin-induced neuropathy in the current investigation to explore the possible mitigative effect of tempol in cisplatin-induced neurotoxicity while addressing the implication of oxidative stress, ER stress, and the autophagy signaling pathway in the therapeutic mechanisms.

The optic nerve is one of the highest oxygen-consuming and energy-demanding tissues in the body. Mitochondria are asymmetrically scattered along optic nerve axons according to local energy requirements (Eells, 2019). Consequently, it is particularly susceptible to neurodegeneration due to energy depletion, ROS accumulation, and mitochondrial dysfunction (Amore et al., 2021). ROS can be counteracted by both enzymatic and non-enzymatic antioxidants. Enzymatic antioxidants include catalase, glutathione peroxidase (GPx), or superoxide dismutase (SOD). SOD is a metalloprotein that catalyzes the dismutation of the superoxide anion radical to molecular oxygen and hydrogen peroxide (H2O2). GPx is an intracellular antioxidant enzyme acting mostly in mitochondria and occasionally in the cytosol (Dong et al., 2006; Pisoschi et al., 2021). Malondialdehyde (MDA) is one of the lipid peroxidation reaction's most assayed end products. It is used as an indicator of oxidative stress (Wadhwa et al., 2012).

The biochemical results of this study revealed a significant increase in the lipid peroxidation marker MDA level and a decrease in GPx and SOD activity in the optic nerve tissues of the CIS-treated group. These data follow the previous studies that signified that oxidative stress is chiefly involved in the pathogenesis of cisplatin-induced optic nerve injury (Icel et al., 2018; Taşli et al., 2018). Moreover, cisplatin-induced DNA adducts may diminish antioxidant enzyme activity, including superoxide dismutase, catalase, and glutathione peroxidase (Yildirim et al., 2022). Pre- and co-treatment with tempol ameliorated cisplatin-induced oxidative stress, which, according to previous studies, supported the antioxidant properties of tempol (Youn et al., 2016; Ewees et al., 2019). Our study's result showed that cisplatin upregulated the mRNA expression levels of Bip, ATF6, and XBP1 besides the proteins of ER stress-induced apoptosis—CHOP and JNK. Former studies demonstrated that ER stress and its mediated apoptosis were linked to cisplatin-induced cytotoxicity in various tissues (Sahu et al., 2019; Huang et al., 2020; Chang et al., 2021). According to Foufelle and Fromenty (2016), ER stress and oxidative stress form a viscous circle. Both can induce each other, which supports our findings. Additionally, inhibition of autophagy worsens ER stress and triggers cell death (Thangaraj et al., 2020). On the other hand, tempol mitigated the ER stress and its mediated apoptosis via the downregulation of their markers. These data are consistent with those reported by Zhang et al. (2019), who verified the ability of tempol to reduce ER stress and its subsequent apoptosis triggered by cadmium in retinal pigment epithelial cells. It is also evident from the current findings that cisplatin inhibited autophagy in rat optic nerves, as the decrease in the mRNA expression levels of Beclin 1 and LC3 was paralleled with an increase in p62 expression. Further confirmation of these findings was provided by the results of immunohistochemical analysis, which indicated the downregulation of LC3 and upregulation of p62 expression. This is in line with previous findings revealing that cisplatin may promote tissue damage by inhibiting autophagy (Zhai et al., 2021; Fu et al., 2022). On the contrary, our results oppose those of Domitrović et al. (2013), who reported that cisplatin activated autophagy while triggering tissue damage. The inhibition of autophagy in this study may be a result of intense stress that overwhelms the cellular adaptive mechanisms comprising autophagy, and these mechanisms were suppressed. Additionally, cytoprotective molecules were converted into cytotoxic ones (Marino et al., 2014). Furthermore, oxidative stress had an inhibitory effect on autophagy by inactivating either autophagy modulators or its core enzymes (Chang et al., 2022), and aberrant ER stress inhibits autophagy in some pathological disorders (Rashid et al., 2015).

Interestingly, autophagy markers were modulated by tempol, as observed from the RT-qPCR and immunohistochemical results denoting autophagy activation that is similar to Ma et al.'s (2017) results, which confirmed the ability of tempol to preserve tissue functions by activating autophagy. In this study, cisplatin-induced histological alterations in the optic nerve include inflammation and apoptosis. Previous studies have reported similar alterations (Icel et al., 2018; Raheem and Mohammed Ali Mahmood, 2023). The current cytological alterations in the optic nerve after cisplatin treatment could be attributed to endoplasmic stress and the inhibition of autophagy. The relationship between ER stress and optic nerve degeneration has been verified by Shimazawa et al. (2012). Moreover, it was suggested that upregulated p62 levels reflected the inhibition of autophagic flux in the optic nerve, resulting in its degeneration (Kitaoka et al., 2020).

The relationship between ER stress and oxidative stress is known to be intertwined, with each being able to induce the other. This concept is supported by the findings of the current study and is in line with the observations of Foufelle and Fromenty (2016). Additionally, the inhibition of autophagy, a cellular process involved in the degradation and recycling of cellular components, has been shown to exacerbate ER stress and promote cell death (Thangaraj et al., 2020). In summary, the results of this study demonstrate that cisplatin-induced optic nerve injury is associated with oxidative stress, ER stress, and apoptosis. The administration of tempol effectively attenuated cisplatin-induced oxidative stress, suggesting its potential as a therapeutic agent for preventing or reducing the neurotoxic effects of cisplatin treatment. These findings contribute to our understanding of the mechanisms underlying cisplatin-induced optic neuropathy and highlight the potential of antioxidant interventions in its management.

Importantly, the inherent limitations of immunohistochemical techniques in precisely identifying the specific cell types marked by antibodies have been reported (Hawes et al., 2009). Immunohistochemistry (IHC) relies on the binding of antibodies to specific antigens within tissues to visualize and identify cellular components (Magaki et al., 2019). While IHC is a valuable tool for examining protein expression and localization, it does not provide definitive proof of cell type identity. To overcome this limitation, by combining multiple approaches, researchers can obtain a more comprehensive and reliable characterization of the cell types present in the examined tissue. Therefore, while IHC provides valuable insights into protein expression patterns within tissues, it is essential to interpret the results cautiously and consider them within the broader context of complementary techniques and knowledge of cell type-specific markers. By employing a combination of methods, researchers can enhance their understanding of cellular identities and provide more accurate interpretations of immunohistochemical data. The ultrastructural examination of the optic nerve of the CIS-treated group in this study revealed several signs of degeneration. Optic nerve fibers were distorted with the disorganization of myelin sheets, as well as the formation of myelin whorls (Buscham et al., 2022). The axonal changes were probably caused by direct damage to retinal ganglion cells or indirect injury to the intraretinal axons (Saggu et al., 2010). Apoptosis is believed to be responsible for the degenerative changes in oligodendrocytes and myelin sheaths of nerve fibers (Oruz et al., 2022). As for the oligodendrocytes and astrocytes, their nuclei were irregular and electron-dense. These morphological alterations in oligodendrocytes indicate impaired oligodendrocyte function. Oxidative stress may be a contributing factor to their damage since oligodendrocytes have low antioxidant defense strategies and high metabolic rates (Narine and Colognato, 2022). Moreover, oligodendrocytes are vulnerable to ER stress, owing to their high metabolic requirements for protein and lipid synthesis (Volpi et al., 2017). In the present study, it was found that pre- and co-treatment with tempol nearly preserved optic nerve architecture in CIS-treated rats (Ewees et al., 2019). Tempol also has been shown to improve the survival of retinal ganglion cells and their axons following injuries induced by partial optic nerve crush and ocular hypertension by inhibiting nitroxidative stress, inflammation, and apoptosis (Yang et al., 2016). Collectively, CIS treatment leads to degenerative changes in the optic nerve, including the distortion and disorganization of myelin, axonal changes, and impaired function of oligodendrocytes. Oxidative stress and ER stress are likely contributing factors to the damage. However, pre- and co-treatment with tempol appears to have a protective effect on the optic nerve architecture and the survival of retinal ganglion cells and their axons in this study and previous research.

Nerve growth factors (NGFs) are essential for neuronal growth, development, and protein synthesis, as well as the stimulation of neurite outgrowth and neurotransmitter synthesis in certain circumstances. They potentially protect against neuron apoptosis (Kolomeyer and Zarbin, 2014). In the current study, the mRNA expression levels of NGF were downregulated in the cisplatin-treated group, and similar findings were reported by Majd et al. (2021). The NGF plays a crucial role in the survival of nerve cells, and its depletion can result in apoptosis (Boia et al., 2020). On the other hand, tempol upregulated NGF mRNA expression levels. The increase in NGF levels may likely be responsible for the improvements in optic nerve histology.

Given the diverse array of cells present within the optic nerve microenvironment, it is imperative to accurately determine the specific cell types in which tempol exerts its effects. To elucidate this, further investigation is required to precisely define the cell types targeted by tempol. Additionally, while it has been identified that tempol influences autophagy and endoplasmic reticulum (ER) stress markers, gaining a deeper understanding of the underlying molecular mechanisms activated by tempol to achieve its biological activity is imperative. Investigating the intricate signaling pathways and molecular interactions involved will provide valuable insights into tempol's mode of action and its therapeutic potential. By employing techniques such as cell-specific markers, transcriptomic analyses, or genetic manipulation, researchers can strive to unravel the specific cell types impacted by tempol within the optic nerve microenvironment. Furthermore, exploring the molecular mechanisms underlying tempol's biological activity, such as its interactions with key signaling pathways or cellular targets, will enhance our understanding of its therapeutic efficacy and optimize its potential clinical applications. Therefore, future comprehensive investigations are warranted to precisely determine the cell types influenced by tempol and to provide a deeper understanding of the molecular mechanisms through which tempol exerts its biological effects in the context of the optic nerve microenvironment.

## 5. Conclusions

As noted in the present study, tempol protected against cisplatin-induced optic neuropathy, and this protection was most likely achieved by the amelioration of oxidative stress and endoplasmic stress as well as the activation of autophagy. Overall, these data provide new insights into the possible neuroprotective mechanisms of tempol against cisplatin-induced optic neuropathy, suggesting that it may be a promising candidate for further clinical studies.

## Data availability statement

The original contributions presented in the study are included in the article/supplementary material, further inquiries can be directed to the corresponding authors.

## Ethics statement

The animal study was approved by Institutional Animal Care and Use Committee (IACUC) of Zagazig University. The study was conducted in accordance with the local legislation and institutional requirements.

## Author contributions

AEA: Conceptualization, Data curation, Formal analysis, Funding acquisition, Investigation, Methodology, Project administration, Resources, Software, Supervision, Validation, Visualization, Writing—original draft, Writing—review and editing. MH: Conceptualization, Investigation, Methodology, Software, Supervision, Writing—review and editing. HM: Conceptualization, Data curation, Formal analysis, Investigation, Methodology, Supervision, Validation, Writing-review and editing. RE: Conceptualization, Data curation, Formal analysis, Investigation, Methodology, Software, Writing—review and editing. IB-D: Conceptualization, Data curation, Investigation, Methodology, Project administration, Software, Writing—review and editing. LF: Conceptualization, Data curation, Formal analysis, Funding acquisition, Investigation, Methodology, Project administration, Resources, Software, Validation, Visualization, Writing—review and editing. MF: Conceptualization, Data curation, Formal analysis, Investigation, Methodology, Resources, Software, Validation, Visualization, Writing—original draft, Writing—review and editing. AHA: Conceptualization, Data curation, Formal analysis, Funding acquisition, Investigation, Methodology, Project administration, Resources, Software, Supervision, Validation, Visualization, Writing—original draft, Writing—review and editing. TK: Conceptualization, Data curation, Formal analysis, Funding acquisition, Investigation, Methodology, Project administration, Resources, Software, Supervision, Validation, Visualization, Writing—original draft, Writing—review and editing.
